# Migration of an ingested orthodontic retainer into the psoas muscle presenting with radiculopathy-like symptoms: A case report

**DOI:** 10.1097/MD.0000000000050811

**Published:** 2026-09-25

**Authors:** Hyun Gu Lee, Hayoung Lee, Youn Young Park

**Affiliations:** a Department of Surgery, Kyung Hee University Hospital at Gangdong, Kyung Hee University College of Medicine, Seoul, South Korea.

**Keywords:** case report, foreign bodies, laparoscopy, psoas muscles, radiculopathy

## Abstract

**Rationale:**

Sharp foreign bodies (FBs) can occasionally perforate the gastrointestinal tract and migrate into adjacent structures, but retroperitoneal migration into the psoas muscle is exceedingly rare. Such cases may present with atypical symptoms and pose diagnostic challenges.

**Patient concerns:**

A 26-year-old man presented with abdominal pain after swallowing an orthodontic retainer. His abdominal pain improved, but he later developed persistent right lower-limb paresthesia and aching pain that worsened with walking.

**Diagnoses:**

Initial computed tomography (CT) demonstrated a metallic FB penetrating a small bowel wall. A diagnostic laparoscopy performed at an outside hospital on day 3 failed to identify the FB. A follow-up CT on day 4 revealed migration of the FB into the right psoas muscle. A repeat CT performed 6 weeks later showed deeper intramuscular migration, correlating with his neurological symptoms.

**Interventions:**

A same-day urgent C-arm–assisted laparoscopic exploration was performed. After right colon mobilization and retroperitoneal exposure, the FB was localized using real-time fluoroscopy and completely removed from the psoas muscle.

**Outcomes:**

The patient’s neurological symptoms improved immediately after surgery, and resolved at the 2-week follow-up. He remained completely asymptomatic at the 12-month postoperative follow-up.

**Lessons:**

Psoas migration of an ingested sharp FB can occur silently without peritonitis and may present with delayed radiculopathy-like symptoms. Persistent or evolving symptoms after an initial negative exploration should prompt repeat CT imaging. C-arm–assisted laparoscopy is a feasible and effective minimally invasive approach for removing migrated retroperitoneal FBs.

## 1. Introduction

Ingestion of foreign bodies (FBs) is a common clinical occurrence. The vast majority (> 80%) of ingested FBs pass spontaneously through the gastrointestinal tract, approximately 10 to 20% require endoscopic retrieval, and fewer than 1% necessitate surgical intervention.^[1–3]^ Sharp or elongated FBs, such as fish bones, needles, or dental appliances, carry a higher risk of gastrointestinal penetration and perforation.

Once perforation occurs, secondary sequelae, such as peritonitis, abscess formation, or fistula, may develop. Occasionally, however, an FB may migrate through the bowel wall into adjacent structures without causing overt peritonitis, a phenomenon sometimes referred to as “silent” or “creeping” perforation.^[4–6]^ Retroperitoneal migration of ingested FBs into structures such as the psoas muscle is exceptionally rare, with only sporadic case reports in the literature.^[7]^ We report a case of a 26-year-old man who swallowed an orthodontic retainer, which subsequently perforated the small bowel and migrated into the right psoas muscle, leading to neurological symptoms mimicking lumbar radiculopathy. This case illustrates a diagnostic pitfall after a negative laparoscopy and demonstrates the value of C-arm–assisted laparoscopy for safe removal.

## 2. Case presentation

A 26-year-old man with no significant medical history presented to the emergency department of an outside hospital with abdominal pain that began after he accidentally swallowed his orthodontic retainer (consisting solely of a stainless steel wire) during sleep (day 0). An initial contrast-enhanced abdominal computed tomography (CT) scan revealed a linear, high-density metallic object consistent with a dental wire, penetrating the wall of an intraperitoneal small bowel loop (proximal jejunum) in the right upper quadrant (Fig. 1A).

**Figure 1.**
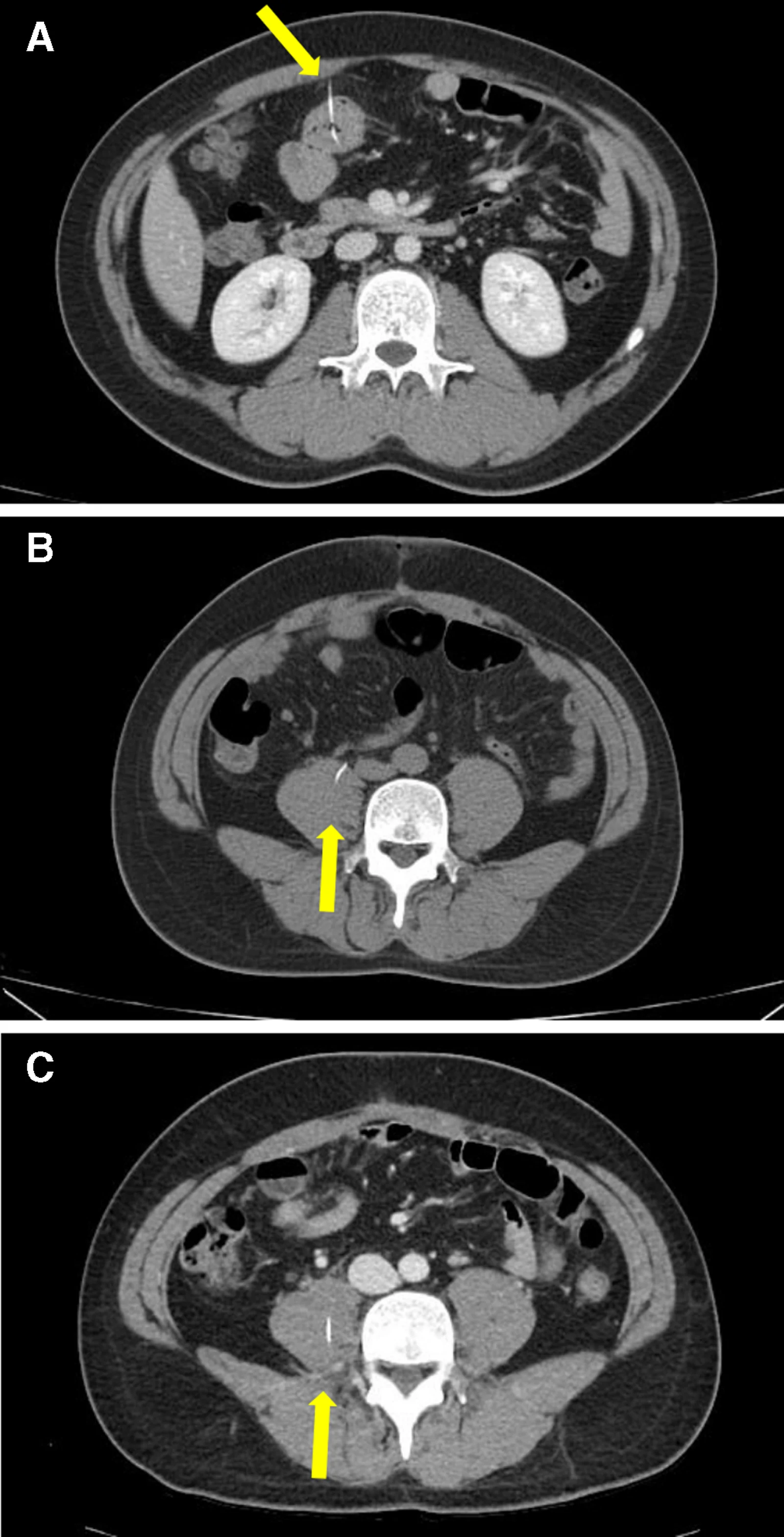
Serial abdominal CT scans demonstrating the migration of the ingested FB. (A) Day 0 contrast-enhanced CT reveals a linear radioopaque object (arrow) penetrating the wall of an intraperitoneal small bowel loop (proximal jejunum) in the right upper quadrant. (B) Day 4 CT shows the FB (arrow) has migrated from the bowel lumen into the right psoas muscle at the L4 to L5 vertebral level, without free air or evidence of peritonitis. (C) Day 43 CT confirms the object (arrow) has migrated deeper into the right psoas muscle. Surrounding soft tissue stranding is consistent with localized chronic inflammatory change. CT = computed tomography, FB = foreign body.

On day 3 post-ingestion, the patient underwent a diagnostic laparoscopic exploration at the outside hospital. However, the FB could not be identified within the peritoneal cavity (negative exploration). Follow-up CT on day 4 showed that the FB had migrated from the bowel lumen into the right psoas muscle at the L4 to L5 vertebral level. No evidence of free air or significant peritonitis was observed (Fig. 1B).

The patient was managed conservatively, and his abdominal pain gradually subsided. However, he subsequently developed a dull, aching pain involving the entire right lower limb, accompanied by intermittent tingling sensations. The discomfort persisted at rest and was exacerbated by prolonged walking or standing.

Approximately 6 weeks (day 43) after the ingestion, he was referred to our institution for further management. On presentation, his vital signs were stable, and an abdominal examination revealed a soft, non-tender abdomen. A neurological examination revealed diffuse paresthesia and aching pain along the right lower limb without motor weakness; an orthopedic consultation was also obtained, which revealed no clinical signs indicative of a primary lumbar radicular syndrome. Because the onset and anatomical localization of symptoms directly corresponded to the FB depth on CT, surgical retrieval was prioritized over spine magnetic resonance imaging or electrophysiological studies. A repeat abdominal CT scan confirmed the presence of the metallic FB, which had migrated even deeper into the right psoas muscle at the L4 to L5 vertebral level, lateral to the aortic bifurcation (Fig. 1C). Given the worsening neurological symptoms and evidence of FB migration, the patient underwent a same-day urgent operation on day 43.

Surgical procedure: the patient was placed in the supine position under general anesthesia. Laparoscopic access was achieved through the previous incision sites. Exploration of the peritoneal cavity was unremarkable, with no evidence of the FB or overt perforation.

The right colon was mobilized by incising the white line of Toldt, thereby exposing the retroperitoneal space. Intraoperative C-arm fluoroscopy localized the metallic FB within the retroperitoneal space, situated between the right ureter and the inferior vena cava, deep within the right psoas muscle (Figs. 2A and 2B).

**Figure 2.**
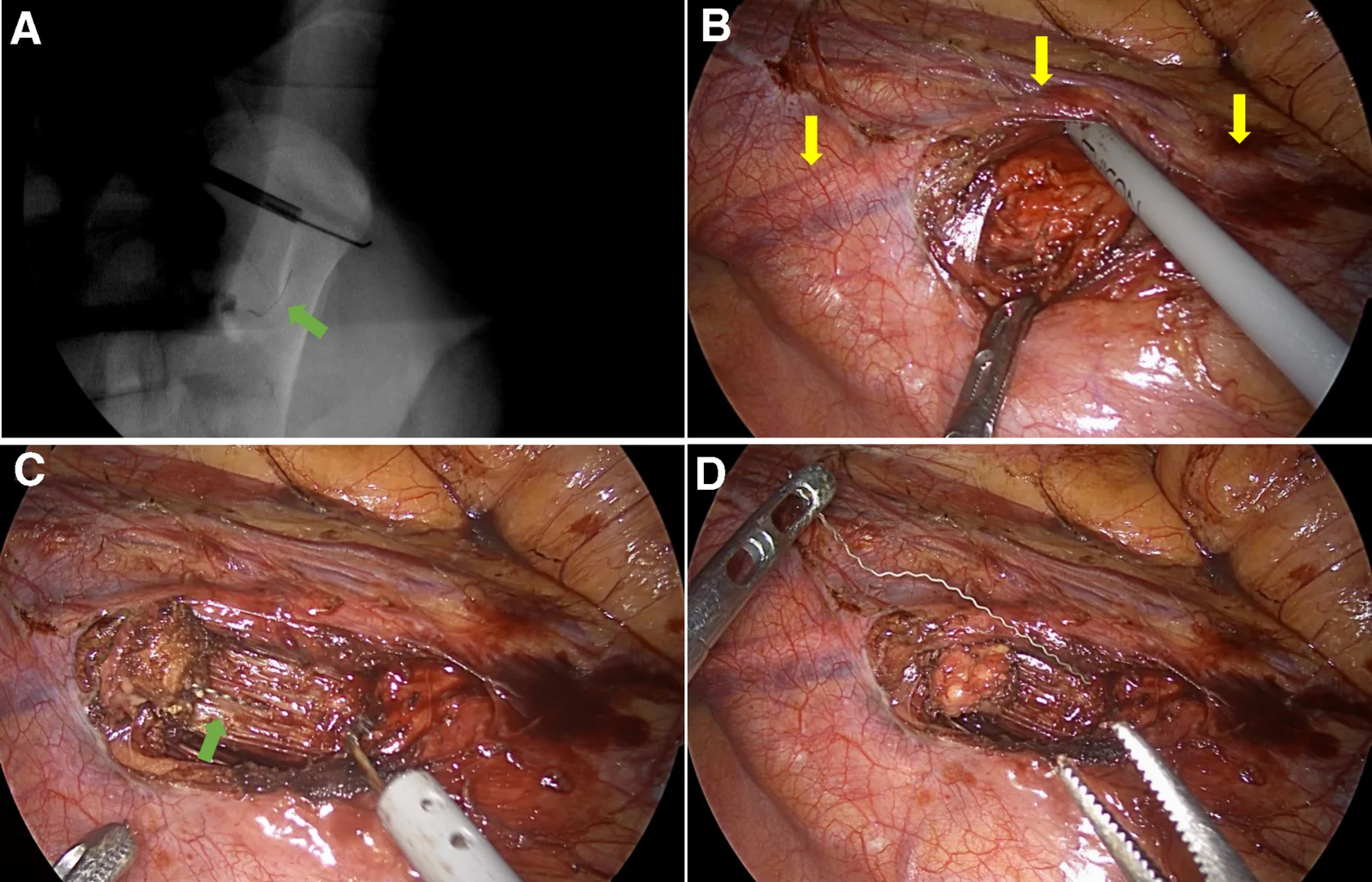
Intraoperative C-arm-assisted laparoscopic localization and removal of the FB. (A) C-arm fluoroscopy localizes the radiopaque FB (green arrow) within the retroperitoneal space, guiding the trajectory of the surgical instrument. (B) Corresponding laparoscopic view during fluoroscopic localization. The mobilized right colon and mesocolon are retracted to expose the retroperitoneum. The course of the right ureter is indicated (arrowheads). (C) Laparoscopic view following dissection of dense fibrotic tissue on the anterior psoas surface, revealing the metallic FB (green arrow) embedded within the muscle fibers. (D) Extracted orthodontic wire retainer after complete removal from the psoas muscle. FB = foreign body.

A dense, fibrotic reaction was noted on the anterior surface of the psoas muscle. This fibrotic tissue was carefully dissected. The metallic wire of the orthodontic retainer was identified embedded within the muscle fibers (Fig. 2C), and it was extracted completely and removed without complication (Fig. 2D). Immediate post-extraction C-arm fluoroscopy confirmed the complete removal of the FB with no residual radiopaque fragments.

Postoperative course: the postoperative course was uneventful. The lower extremity paresthesia and pain improved immediately after the removal of the FB. He resumed a soft diet on postoperative day 1 and was discharged home on postoperative day 2. At the 2-week outpatient follow-up, he reported resolution of all neurological symptoms and expressed satisfaction with the minimally invasive surgical outcome and rapid recovery; no further routine hospital visits were required. At a 12-month postoperative telephonic follow-up, the patient confirmed that he remained completely asymptomatic without recurrence of lower-limb discomfort. The timeline of the patient’s clinical presentation, diagnostic findings, and treatment process is summarized in Table 1.

**Table 1:** Timeline of clinical events and patient care.

Time point	Clinical events and interventions	Diagnostic imaging & findings
Day 0	Accidental ingestion of orthodontic retainer during sleep; onset of abdominal pain. Presenting to an outside hospital emergency department.	Initial abdominal CT: linear metallic FB penetrating an intraperitoneal small bowel loop (proximal jejunum) in the RUQ.
Day 3	Diagnostic laparoscopy at outside hospital. No FB identified in the peritoneal cavity (negative exploration).	
Day 4	Abdominal pain gradually subsides; conservative management.	Follow-up CT: FB migrated from bowel lumen into the right psoas muscle at the L4 to L5 vertebral level (no free air or peritonitis).
Day 4–42	Onset of persistent right lower-limb paresthesia and aching pain (lumbar radiculopathy-like symptoms) worsening with walking.	
Day 43	Referral to our institution. Orthopedic and neurological examination performed. Same-day urgent C-arm-assisted laparoscopic retroperitoneal exploration. Complete extraction of metallic wire from right psoas.	Repeat CT: deeper migration into right psoas muscle at L4 to L5 level. Intraoperative C-arm & Post-extraction fluoroscopy: Confirmed complete retrieval with no residual fragments.
Day 44 (POD 1)	Neuropathic symptoms improved immediately. Resumed soft diet.	
Day 45 (POD 2)	Discharged home without complications.	
Day 57 (POD 14)	Outpatient visit: Resolution of all lower-limb neuropathic symptoms. Routine hospital follow-up completed.	
Day 408 (postoperative month 12)	Telephonic follow-up	Patient reported remaining symptom-free with no recurrence of lower-limb pain or paresthesia.

CT = computed tomography, FB = foreign body, POD = postoperative day, RUQ = right upper quadrant.

## 3. Discussion

We have presented an exceedingly rare case of an ingested orthodontic retainer that perforated the small bowel, migrated into the retroperitoneal psoas muscle, and presented with neurological symptoms mimicking lumbar radiculopathy. This case is clinically instructive for 2 primary reasons: first, it involves an an extremely unusual migratory path from an intraperitoneal organ (small bowel) to a deep retroperitoneal structure, and second, the patient’s presentation was dominated by delayed-onset neurological symptoms, not the expected signs of peritonitis, which posed a significant diagnostic challenge.

FB migration into the psoas muscle is most commonly reported following perforation of primarily retroperitoneal organs, such as the duodenum, appendix, or the ascending or descending colon.^[8,9]^ Although retroperitoneal organ perforation provides a direct path to the psoas, reevaluation of initial CTs in our patient confirmed the origin in a mobile intraperitoneal jejunal loop. Migration from an intraperitoneal small bowel loop without peritonitis, as seen in our patient, is exceptionally rare. The pathophysiology of silent or creeping perforation (where the FB penetrates the bowel wall without causing overt peritonitis) is thought to involve contained injury, fibrotic reaction, and absence of free air or significant peritoneal contamination.^[10]^ In our case, the initial rapid descent of the FB to the L4 to L5 level likely reflects the natural intraperitoneal mobility of the affected jejunum prior to retroperitoneal anchoring. Following this, a plausible mechanism involves localized inflammatory adhesion of the perforated jejunal loop to the posterior parietal peritoneum. Subsequent intestinal peristalsis and body movements likely exerted mechanical pressure on the sharp wire, driving its gradual, “creeping” penetration through the parietal peritoneum into the retroperitoneal psoas muscle, while localized fibrosis effectively sealed the perforation site.

The unique feature of this case was the transition from transient abdominal pain to persistent neuropathic leg symptoms. The lumbar plexus forms within the psoas muscle. Consequently, any intramuscular lesion, such as an abscess, hematoma, or, in this instance, a metallic FB with chronic inflammation, can irritate or compress its neural components, producing femoral neuropathy or plexopathy.^[11,12]^ This anatomic relationship explains the patient’s diffuse paresthesia and aching pain radiating throughout the right leg. Such symptoms can closely mimic common spinal or musculoskeletal disorders, including lumbar disc herniation, which may lead to delayed recognition of the underlying cause.

The initial negative laparoscopy at the outside hospital represents an important diagnostic pitfall. By day 3, the FB had likely already traversed the bowel wall and become sequestered within the retroperitoneal space. Since the exploration was confined to the intraperitoneal cavity, the migrated FB was invisible to inspection. This sequence illustrates how “silent” perforation can mislead clinicians when follow-up imaging is not performed promptly. The follow-up CT on day 4 proved decisive, revealing the FB’s new retroperitoneal location and confirming that migration had occurred. These findings reinforce the need for serial CT assessment in patients whose symptoms persist or evolve after initial management, even when intra-abdominal findings appear benign.

The management of FB migration into the psoas muscle is not standardized due to the rarity of such cases. Conservative management may be considered in asymptomatic patients, but surgical intervention is warranted in cases with persistent symptoms, neurological deficits, or risk of infection. In this case, timely reevaluation and the combined use of laparoscopy and intraoperative C-arm fluoroscopy allowed precise localization and safe removal of the metallic object. This hybrid technique, which has been successfully utilized for other complex abdominal FBs, allowed precise localization and safe, minimally invasive extraction.^[13]^ Compared with traditional open exploration, it may offer excellent visualization, complete retrieval with minimal tissue injury, and rapid postoperative recovery, underscoring its potential value in managing similar complex presentations.

Strengths of this report include the detailed documentation of radiologic findings and surgical technique. Limitations include the single-case nature of the report and the absence of preoperative lumbar spine magnetic resonance imaging or electrophysiological studies. Furthermore, while a telephonic follow-up at 12 months postoperatively confirmed that the patient remained completely symptom-free, the reliance on telephonic inquiry rather than in-person clinical examination or follow-up imaging beyond the initial 2-week postoperative visit represents a methodological limitation.

## 4. Conclusion

This case reports an extremely rare complication of FB ingestion with an unusual migratory path and clinical presentation. It highlights the potential for sharp FBs to migrate to unexpected locations and underscores the importance of follow-up CT imaging after a negative exploration. Furthermore, a C-arm-assisted laparoscopic approach is a feasible and safe strategy for the management of such retroperitoneal cases.

## Author contributions

**Conceptualization:** Hyun Gu Lee, Youn Young Park.

**Data curation:** Hyun Gu Lee.

**Investigation:** Hyun Gu Lee, Hayoung Lee, Youn Young Park.

**Supervision:** Youn Young Park.

**Writing – original draft:** Hyun Gu Lee.

**Writing – review & editing:** Hayoung Lee, Youn Young Park.
